# *Mycoplasma pneumonia:* Clinical features and management

**DOI:** 10.4103/0970-2113.63611

**Published:** 2010

**Authors:** Surender Kashyap, Malay Sarkar

**Affiliations:** *Department of Pulmonary, Indira Gandhi Medical College, Shimla, India*

**Keywords:** Asthma, macrolides, mollicuites mycoplasma, serology

## Abstract

*Mycoplasma pneumonia* is a common respiratory pathogen that produces diseases of varied severity ranging from mild upper respiratory tract infection to severe atypical pneumonia. Apart from respiratory tract infections, this organism is also responsible for producing a wide spectrum of non-pulmonary manifestations including neurological, hepatic, cardiac diseases, hemolytic anemia, polyarthritis and erythema multiforme. This review focuses on molecular taxonomy, biological characteristics, epidemiology, clinical presentation, radiology and various laboratory tools in diagnosis, differential diagnosis, treatment and prevention of mycoplasma pneumonia.

## INTRODUCTION

*Mycoplasma pneumonia* is a common respiratory pathogen that produces diseases of varied severity ranging from mild upper respiratory tract infection to severe atypical pneumonia. Although rarely fatal, *M. pneumoniae* is an important cause of acute respiratory tract infection, especially as a potential etiology of the clinical entity termed "atypical pneumonia". Initially it was known as Eaton agent, after Eaton *et al*, identified this plero-pulmonary like organisms from the sputum of patients with primary atypical pneumonia in 1944. It subsequently renamed as Mycoplasma. Of the many species of Mycoplasma known to infect man, *M. pneumoniae* is an important cause of respiratory tract infections. Apart from respiratory tract infections, this organism is also responsible for producing a wide spectrum of non-pulmonary manifestations including neurological, hepatic, cardiac diseases, hemolytic anemia, polyarthritis and erythema multiforme. Of the non-pulmonary manifestation, neurological manifestations are thought to be the most common.[1]

## MOLECULAR TAXONOMY AND BIOLOGICAL CHARACTERISTICS

Mycoplasma is the smallest and simplest self limiting bacteria.[1] It belongs to the class Mollicutes and family Mycoplasmataceae. Mycoplasmas are distinguished from bacteria by the lack of cell wall structure. The absence of cell wall structure makes these organisms insensitive to beta-lactam anti microbial agents, prevents them from staining by gram's stain, and is largely responsible for their polymorphism. Mycoplasmas have an extremely small[2] genome (0.58 to 2.20Mb) compared with the 4.64Mb of *E. coli*. Maniloff[3] proposed that the small genome size is due to gradual reduction in genome size from a common gram positive ancestor by the process of degenerative evolution. So phylogenetically, Mycoplasmas are more closely related to gram positive bacteria. The extremely small size (150-200 nm) and its limited metabolic and biosynthetic capabilities are responsible for many of the biological characteristics of the organisms. It explains the parasitic and saprophytic existence of the organisms and its fastidious growth requirements which may complicate its detection by culture. Mycoplasma reproduces by binary fission.

It is an extra cellular pathogen, its survival depends on adherence to the respiratory epithelium, and this fixation to ciliary membranes is primarily by interactive adhesion and accessory proteins. The major adhesion is P1 adhesin. Cytoadhesion protects mycoplasmas from mucociliary clearance. Hydrogen peroxide is produced locally, which has a cytopathic effect on airway epithelium and cilia and is responsible for persistent cough. Mycoplasma is also being incriminated in the development of autoimmunity which can explain the extra pulmonary involvement. Production of multiple arrays of cytokines and other reactive substances in the pathogenesis[4] of *M. pneumoniae* lung disease has been a subject of considerable interest during past several years.

Current evidence from human and animal studies suggests that cytokine production and lymphocyte activation may either minimize disease through the enhancement of host defense mechanisms or exacerbate disease through immunological lesion development. The more vigorous the cell-mediated immune response and cytokine stimulation, more severe is the clinical illness and pulmonary injury. This concept of immune- mediated lung disease provides a basis for consideration of immuno-modulatory therapeutics in addition to conventional antimicrobial therapies. Immunity to mycoplasma is transient and recurrences are frequent

## EPIDEMIOLOGY

*M. pneumoniae* infections can involve both the upper and lower respiratory tract and occurred worldwide in a endemic fashion with epidemic spurt at four to seven year intervals.[5] Climate, seasonality, and geography are not thought to be of major significance, although most outbreaks in USA[6] tended to occur during late summer and early fall. On the basis of serological studies, Lind *et al*,[7] showed a pattern of endemic disease transmission over a period of 50 years, punctuated with cyclic epidemics every three to five years. The long incubation period, relatively low transmission rate, and persistence of organisms in respiratory tract for variable periods following infections might explain the prolonged duration of epidemics. Dorigo-Zetsma [8] *et al*, genotyped *M. pneumoniae* clinical isolates and grouped them into eight subtypes within two genomic groups based on P1 adhesin subtypes. Different P1 adhesin subtypes may be operating in the development and cycling times of *M. pneumoniae* epidemiology. Such gene divergences within the P1 adhesin and development of subtype specific antibodies, following initial infection, might account for the frequency of re-infection, which may be due to another subtype.[9] Infection is transmitted via aerosols from person to person. Since the organisms tend to be associated with desquamated cells, relatively large droplets may be required for transmission as evidenced by close personal contact typical of outbreak settings-schools, military barracks, and institution. In view of the intimate contact needed for droplet transmission and the slow (six-hour) generation time of *M. pneumoniae*, one to three weeks of incubation period is typical for each case.

Although, *M. pneumoniae* is a well recognized pulmonary pathogen in the West, information on disease prevalence in our country is sparse due to non-availability of reliable, rapid diagnostic techniques as well as the lack of clinical awareness. A study[10] at a tertiary care center at Delhi reported *M. pneumoniae* infection in 18(24%) of 75 children with CAP using the criteria of culture and/or serology and/or a positive PCR assay on nasopharyngeal aspirate.

We reported [11] *M. pneumoniae* infections based on serology in 15% of CAP in adults. Similarly, Shenoy *et al*.[12] report that *M. pneumoniae* was responsible for 24% cases of pneumonia in hospitalized children. High prevalence of *Mycoplasma pneumoniae* infection was reported by Chaudhry *et al*,[13] among children with community acquired pneumonia with serological positivity of 27.4%.

## CLINICAL PRESENTATION

*M. pneumoniae* causes up to 40% or more of community acquired pneumonia (CAP) cases and as many as 18% of cases requiring hospitalizations in children.[14] Older studies reported *M. pneumoniae* pneumonia to be somewhat uncommon in children aged less than five years and greatest among school aged children 5-15 years of age with decline after adolescence and on into adulthood.[15] However, the latter studies have documented that *M. pneumoniae* may occur endemically and occasionally epidemically in older persons, as well as in children under five years of age. These findings may reflect improved detection abilities that were unavailable in the 1960s and 1970s when the first epidemiological descriptions of *M. pneumoniae* were published.

The clinical presentation of *M. pneumoniae* respiratory disease is often similar to what is seen with other atypical pathogens, particularly *Chlamydia pneumoniae*, various respiratory viruses and bacteria. *M. pneumoniae* may[14] also be present in the respiratory tract concomitantly with other pathogens and there is some evidence from humans and animal models indicating that infection with *M. pneumoniae* may precede and somehow intensify subsequent infections with various respiratory viruses[16] and bacteria including *S. pyogenes* and *Neisseria meningitides*. Potential explanation for such a synergistic effect includes immunosuppression or alteration in respiratory tract flora due to the presence of *M. pneumoniae*. Similarly, in a prospective cross-sectional study Dey *et al*,[17] report a 35% prevalence of Mycoplasma infection among patients diagnosed with community acquired pneumonia. They also detected secondary bacterial infection in a large number of patients. In patients with *Mycoplasma pneumoniae* infection, they isolated other bacteria from blood in 50% cases and from respiratory tract secretions in 68% cases. Therefore, initial empirical antibiotic therapy for community acquired pneumonia in India must include antibiotics with activity against *M. pneumoniae*. Staugas *et al*.[18] also reported high prevalence of secondary bacterial infection. They proposed that *M. pneumoniae* by penetrating mucociliary blanket caused epithelial cell damage and also reduced mucociliary clearance. If it was not detected early, it may lead to development of pulmonary sequelae.

*M. pneumoniae* is ordinarily mild, and many adult cases may be asymptomatic, whereas this is much less common in children, perhaps reflecting some degree of protective immunity for reinfections. Although most infections occur among outpatients (hence the colloquial term walking pneumonia), *M. pneumonia* is a significant cause of bacterial pneumonia in adults requiring hospitalization in USA. Marston *et al*.[19] report that *M. pneumoniae* was definitively responsible for 5.4% and possibly responsible for 32.5% of 2776 cases of CAP in hospitalized adults based on complement fixation (CF) test for detection of infections. An additional striking finding of this study was their observation that the incidence of pneumonia due to *M. pneumoniae* in hospitalized adults increased with age and it was second only to *S. pneumoniae* in elderly persons.

*M. pneumoniae* can affect the upper or lower respiratory tracts or both. Symptoms commonly appear gradually, during a few days, and can persist for weeks or months. Typical clinical features[20] include an initial pharyngitis, sore throat and hoarseness, fever. An intractable day and night cough characterizes extension of the infection to the lower airways. Initially cough is non-productive but later may yield small to moderate amounts of non-bloody sputum. Dyspnoea may be evident in more severe cases.

Children[21] under five years of age are most likely to manifest coryza and wheezing, and progression to pneumonia is relatively uncommon, whereas older children aged 5-15 years are more likely to develop bronchopneumonia in one or more lobes, even sometimes requiring hospitalizations. Mild infections and asymptomatic conditions are particularly common in adults, and bronchopneumonia develops in 3-10% of infected persons.

Chest auscultation may show scattered or localized rhonchi and expiratory wheezes. Since alveoli are usually spared, rales and frank consolidation are fairly uncommon unless atelectasis is widespread. In uncomplicated cases, the acute febrile period lasts about a week, while the cough and lassitude may last two weeks or even longer. The duration of symptoms and signs will generally be shorter if antibiotics are started early in the course of illness. Though there are a few reports of Mycoplasma infections in HIV positive patients, it is not known whether incidence, severity of Mycoplasma infections or host response to Mycoplasma infection is altered by immunosuppression. There are a few case reports of fulminant[22,23] plasma infection with multiple organ involvement but otherwise this is uncommon. Predisposing factors include sickle cell related hemoglobinopathies and hypo gammaglobulinemia, Downs syndrome and various immunosuppressive states but fulminant course may also occur in previously healthy persons, particularly in young males and smokers.

Extra pulmonary manifestations during *M. pneumoniae* infections may sometimes overshadow the respiratory picture. Presence of multiple extra pulmonary manifestations is an ominous[24] prognostic factor. As many as 25% of persons infected with *M. pneumoniae* may experience extra pulmonary complications at variable time periods after onset of or even in the absence of respiratory illness. Immunopathogenetic factors are probably involved in many of the extra pulmonary complications given the cross reactivity between human and *M. pneumoniae* antigens. Direct invasion should also be considered as *M. pneumoniae* has been detected in extra pulmonary sites by culture and PCR testing. It is also important to realize that extra pulmonary complications can be seen before, during, or after pulmonary manifestations or even can occur in the complete absence of any respiratory symptoms. Extra pulmonary[4] manifestations may occur not less than three days after the onset of respiratory disease; and for two to three weeks after the respiratory disease has resolved.

Central nervous system (CNS) manifestations are the most frequent extra-pulmonary complications of *M. pneumoniae* infection and can at times be life threatening.[25]

Encephalitis and meningoencephalitis [26] are most commonly followed by polyradiculitis and aseptic meningitis. Frequently, a manifest respiratory infection precedes the CNS symptoms. The mean interval between the onset of respiratory symptoms and CNS manifestations was 9.6 days (range 2-14 days) in the study by Tsiodras *et al*.[27] *M. pneumoniae* infection should be routinely considered in the differential diagnosis of patients with CNS manifestations, especially if associated with pneumonia. Among serologically confirmed *M. pneumoniae* infections that require hospitalizations, 1-10%[28] are associated with neurological manifestations. The overall incidence is<0.1%, although the exact incidence of *M. pneumoniae*-associated CNS complications remains unknown due to the absence of an appropriate diagnostic test. It is suggested that the complications may result either from direct invasion of *M. pneumoniae* into the brain, a neurotoxin produced by the organism or an immune-mediated damage. The immune-mediated injury could be caused by cross-reacting antibodies to antigen(s) shared by Mycoplasma and brain, organism induced immunosuppression, immune complex vasculopathy, or vascular micro thrombi.[26,29]

### Skin and mucosal manifestations

Among patients with *M. pneumoniae* infection, 25% may have dermatological manifestations, making these one of the common [28] complications of this infection. There is a well-known association between Mycoplasma and Stevens-Johnson syndrome, erythema multiforme and toxic epidermal necrolysis. *M. pneumoniae* is the most common infectious[30] agent associated with Stevens-Johnson syndrome. The Stevens-Johnson [31] syndrome (SJS) is an acute, self-limited disease characterized by severe inflammation and necrosis of two or more mucous membranes with systemic symptoms such as fever and malaise. This is in contrast to erythema multiforme (EM), which is a mild cutaneous illness characterized by symmetrically distributed, fixed, discrete, round, red papules, some of which evolve into target lesions, but without mucosal involvement and systemic toxicity.

Some cases of Stevens-Johnson syndrome have been reported to affect mucosal membranes exclusively, leaving the skin intact. It is unclear at present whether this entity is a variant of Stevens-Johnson syndrome or a new entity. Latsch *et al*.[32] described it as atypical Stevens-Johnson syndrome. Patients with oral, as well as genitourinary, mucosal lesions generally manifest with fever and generalized fatigue. Antimicrobial therapy rapidly resolves the clinical condition. It can also cause urticaria, toxic epidermal necrolysis and pityriasis rosea. The exact mechanism of skin and mucosal disease is unknown, but immune complex-mediated vascular injury, cell-mediated immune response and cytotoxic injury to epithelial cells, and autoimmune mechanisms have been suggested.

### Hematological manifestations

Hematological manifestations of *M. pneumoniae* infection include autoimmune hemolytic anemia, autoimmune thrombocytopenia and disseminated intravascular coagulation. The mechanism may involve cross-reaction with cold agglutinins.[20] Cold antibody formation is a well known feature following *M. pneumoniae* infection. The predominant type of cold antibody seen following this infection is anti-I. Corticosteroids are used in the treatment of severe hemolytic anemia due to *M. pneumoniae*. Blood transfusions too will be required rarely. *M. pneumoniae* may occasionally cause unusual and severe complication like hemo phagocytic syndrome.[33] This syndrome is characterized by a systemic activation of macrophages/histiocytes which are induced to undergo phagocytosis of hematopoietic elements.

### Gastrointestinal manifestations

Gastrointestinal manifestations are frequent[20,34] and have been described roughly in 25% of cases, manifesting as nausea, vomiting, abdominal pain, diarrhea and loss of appetite. Cholestatic hepatitis and pancreatitis, albeit rare, can occur. Most case reports showed that liver involvement of *M. pneumoniae* infection is cholestasis rather than hepatic necrosis. Acute fulminant hepatic failure due to mycoplasma is a rare manifestation. There has been one case report[35] of multiple hypoechoic lesion in spleen associated with mycoplasma infection.

### Musculo-skeletal manifestations

*M. pneumoniae* infection is associated with non-specific myalgias, arthralgias and polyarthropathies in approximately 14% of cases,[20] with complete recuperation during disease evolution; but they can persist for long periods.

Polyarthritis of mycoplasma origin could mimic acute rheumatic fever. Unlike in rheumatic fever,[34] polyarthritis of mycoplasma origin is usually associated with a moderately high ESR and there is no neutrophil leucocytosis.

### Renal manifestations

Glomerulonephritis associated with *M. pneumoniae* is rare, and a few cases have been described in children. Clinical manifestations may be due to acute infection of the kidney and related structures or due to an immunological process. Renal manifestations include progressive glomerulonephritis, nephrotic syndrome, transient massive proteinuria, chronic renal failure due to cold agglutinin, acute interstitial nephritis, acute renal failure due to acute nephritis, hemoglobinuria or hemolytic uremic syndrome, isolated hematuria, cystitis or urethritis. The most frequent lesion is membranoprofilerative glomerulonephritis.[20,34] Other extra-pulmonary manifestations are presented in Table 1.

**Table 1:** Extra pulmonary manifestations of *M. pneumoniae* infection[4,26‐29,36‐39]

Neurological manifestations	Aseptic meningitis
	Meningo encephalitis
	Cerebro vascular accidents
	Hemiplegia
	Transeverse myelitis, ascending paralysis
	Cranial nerve palsy, cerebellar atxia
	Optic neuritis, polyradiculopathy
	Peripheral neuropathy
	Guillain‐Barr'e syndrome
Musculoskeletal	Arthralgias myalgias
	Septic arthritis,
	Polyarthritis
	Acute rhabdomyolysis
Hematological	Hemolytic anemia
	Thrombotic thrombocytopenia purpura
	Intravascular coagulation
	Hemophagocytic syndrome
Cardiovascular	Pericarditis, myocarditis
	Endocarditis, CCF
	Pericardial effusion
	Raynaud's phenomenon
Dermatological	Skin rashes
	Stevens‐Johnson syndrome
	Erythemanodosum
	Bullous erythem a multiforme
Gastrointestinal	Diarrhea, pancreatitis
	Cholestatic hepatitis
	Hypoechoic lesions in spleen
Renal	Acute glomerulonephritis, IgA nephropathy
	Tubulointerstitial nephritis, renal failure

*M. pneumoniae* and *C. pneumoniae* closely resemble each other. However, there are certain distinguishing [39] features that may help in predicting the etiological agent.

1. *M. pneumoniae* is an acute infectious disease, while in contrast *C. pneumoniae* may be acute but is typically a chronic disease.
2. *M. pneumoniae* has a predilection for both the upper and, as well as, lower respiratory tract. Thus patients with CAP presenting with upper respiratory tract involvement are most likely to have *M. pneumoniae*. Common upper respiratory tract manifestations in a patient with CAP include otitis, bullous myringitis, and mild non exudative pharyngitis. These findings are less common with *C. pneumoniae* CAP.
3. Laryngitis is the most important clinical finding to differentiate the Mycoplasma from *C. pneumoniae*. Athough all patients with *C. pneumoniae* CAP do not have laryngitis, the majority of them do. Patients presenting with a Mycoplasma like illness with pneumonia-associated hoarseness should be considered as having *C. pneumoniae* until proven otherwise.
4. Gastrointestinal involvement is typical for Mycoplasma, and is much less common with *C. pneumoniae* pneumonia.

Puljiz[40] *et al*. reported that the incidence of cough was higher among Mycoplasma group. They also reported significant elevation of C-reactive protein (CRP) and aspartate aminotransferase (AST) level among *C .pneumoniae* group than in *M. pneumoniae* group. The reason is that *C. pneumoniae* invades the blood and spreads into different organs, while *M. pneumoniae* remains on the respiratory tract epithelium causing a weaker inflammatory reaction with lower values of CRP and AST.

Following clinical features may be helpful in differentiating[39] Legionella and *Mycoplasma pneumoniae* pneumonia. Relative bradycardia is a constant clinical sign of Legionella pneumonia, but not in case of *M. pneumoniae* pneumonia. If a patient with CAP[39] has abdominal pain with or without loose stools or diarrhea, then Legionella is highly likely since no other cause of CAP is associated with acute abdominal pain. Upper respiratory tract [39] involvement, erythema multiforme favor infection with *M. pneumoniae* while hepatic involvement and electrolytic abnormalities (hyponatremia and hypophosphatemia) are characteristic features of Legionella pneumonia.

### Differential diagnosis between *M. pneumoniae* pneumonia and other bacterial pneumonia

The Japanese Respiratory Society (JRS) proposed a scoring system to differentiate between atypical and bacterial pneumonia.[41] The guidelines set up six parameters based on clinical symptoms, physical signs, and laboratory data. These parameters are; 1) under 60 years of age 2) no or minor comorbid illness 3) the patient has stubborn cough 4) Poor chest auscultatory findings 5) no sputum or identified etiologic agent by rapid diagnostic tests, and 6) a peripheral white blood cells count below 10,000/cmm. When there is a correlation of items of more than four parameters among all six parameters, then the guidelines recommend the use of macrolides or tetracyclines for a suspected atypical pneumonia. If these criteria are not met, the guidelines recommend the use of beta lactams for suspected bacterial pneumonia. This differentiation assumes significance in areas where incidence of macrolide resistant is high e.g. in Japan.

## EFFECT ON ASTHMA

The role of atypical pathogens- C. *pneumoniae* and *M. pneumoniae* in asthma has become an active area of investigation in the recent years.[42] *M. pneumoniae*, primarily considered a causative agent of CAP has recently been linked to asthma in various ways. Infection with this organism may precede the onset of asthma. Secondly, it may exacerbate asthma and make control of asthma more difficult. In a serological based study involving four atypical pathogens-*C. pneumoniae, M. pneumoniae*, Legionella, and *C. burnettii*, Lieberman *et al*,[43] found that only infection with *M. pneumoniae* is associated with hospitalization for acute exacerbation of bronchial asthma. In most of these *M. pneumoniae* infected patients, there is also evidence of infection with respiratory virus. However, the pathophysiological and therapeutic significance of these findings need to be confirmed in proper design trials. Biscardi[44] *et al*. report that *M. pneumoniae* was a causative microbe in 20% of exacerbations in asthmatic children requiring hospitalization for asthma exacerbations. This high rate was not confined to previously diagnosed asthmatics. Fifty per cent of children experiencing their first asthma attack were also positive for *M. pneumoniae*. These figures were significantly greater than for other bacteria or viruses that were evaluated in this study. Sixty two percent of first time asthmatic patients who were positive for *M. pneumoniae* or *C. pneumoniae* had recurrent asthma episodes, whereas only 27% of pathogen-free patients had a subsequent attack (*P*-value< 0.05). Thus, *M. pneumoniae* seems to play an important role both in index and subsequent asthma exacerbations.

Martin [45] *et al*. conducted a bronchoscopy based study to determine the prevalence of *M. pneumoniae* or *C. pneumoniae* in the airways of chronic stable asthmatics. They found atypical bacteria in 56% of chronic, stable asthmatics who had experienced no exacerbations within 3 months of enrollment. Twenty three had *M. pneumoniae*, two patients had other pathogenic mycoplasma but only one control had mycoplasma in the airways. Thus, there was a highly significant difference between the asthmatics and controls (*P*=0.007). In a double-blind treatment trial, Kraft[46] *et al*, demonstrate that receiving Clarithromycin improved FEV1 only in asthmatics that were positive for *M. pneumoniae* or *C. pneumoniae* in their airways. The FEV1 did not improve in asthmatics who did not have these bacteria in their airways and who received clarithromycin. However, the exact mechanism of macrolides in reducing asthma symptoms in *M. pneumoniae* infected patients, needs to be elucidated before developing any firm conclusion as macrolides may improve lung function by both anti microbial and immuno modulatory effect. We have limited data regarding prevalence and association of *Mycoplasma pneumoniae* respiratory infection with asthma in our country. Recently, Chaudhry[47] *et al*. found a statistically significant association between *Mycoplasma pneumoniae* infection and children having moderate and severe persistence asthma. They also showed a significant association of *Mycoplasma pneumoniae* infection with acute exacerbation of asthma in previously diagnosed asthmatics children.

### Pathophysiological mechanisms

Effects of mycoplasmal infections on airways seem to be multifactorial, and involve a complex interplay of airway inflammation and IgE mediated hypersensitivity, in addition to features of individual patients such as atopic predisposition.[42]

1. Mycoplasma infections may result in T-helper type 2 predominant airways disease and development of airway inflammation that may induce or exacerbate asthma.
2. IgE related hypersensitivity

## RADIOLOGY

Radiographic manifestations of *M. pneumoniae* pneumonia can be extremely variable and can mimic a wide variety of lung diseases. The inflammatory response causes interstitial mononuclear cell inflammation that may be manifested radiographically as diffuse, reticular infiltrates of bronchopneumonia in the perihilar regions or lower lobes, usually with a unilateral distribution, and hilar adenopathy. Bilateral involvement may occur in about 20% of cases. Another study by Puljiz *et al*.[40] found that the commonest chest x-ray abnormality was interstitial infiltrate in 90.48% followed by alveolar infiltrates in 8.84% cases. Pleural effusion was detected in 13 patients (8.84%).

In a retrospective review of chest CT between mycoplasma and other CAP patients, Takahito [48] *et al*. found that bronchial wall thickening (*P* value< 0.0001) was the commonest abnormality in *M. pneumoniae* group. *M. pneumoniae* attaches to cilia via P1 protein and multiplies in the respiratory epithelial layer. Attachment to epithelial cilia is responsible for bronchial wall thickenings.

## LABORATORY DIAGNOSIS

Diagnosis of *M. pneumoniae* infection is challenging due to the fastidious nature of the pathogen, the considerable sero-prevalence, and the possibility of transient asymptomatic carriage. Lab diagnosis is greatly hampered by the lack of standardized, sensitive and specific methods [49] for the detection of this atypical respiratory pathogen. A second major barrier is the difficulty in sampling the lower respiratory tract infection in representative populations of patients. However, a specific diagnosis is important because beta-lactam antibiotics used empirically in the treatment of community acquired pneumonia are ineffective against *M. pneumoniae*.

Cultures can provide information regarding viability and biological characteristics of Mycoplasma, and anti-microbial susceptibility testing as well as assessing microbiological efficacy in treatment trials. But the main hindrances for successful cultures are requirement of specialized techniques due to the fastidious growth requirement, proper specimens processing and many days for growth detection. Moreover, culture is successful in only 30-60% of the serologically diagnosed[50] cases. So serological testing is the most common mean of diagnosing *M. pneumoniae* infection. Serological tests are easy to perform but it is not without flaws. They are generally non-specific and retrospective in nature. It needs convalescent serum specimens to show sero-conversions or a fourfold increase in titer. Nevertheless, it is the most useful means of determining the cause of an outbreak or the prevalence of infections in epidemiological studies and a four-fold rise in titer between acute and convalescent sera is still considered a "gold standard" to diagnose acute *M. pneumoniae* respiratory [51] infections

## SEROLOGY

Before the availability of more advanced serological techniques, detection of cold agglutinins was considered a valuable tool to diagnose *M. pneumoniae* infection. The formation of cold agglutinins is the first humoral immune response to *M. pneumoniae* infection. Cold agglutinins usually appear by the end of the first week or the beginning of the second week of illness and disappear by 2-3 months.[52] Determination of these auto-antibodies is first and simple to perform but they are not very reliable indicators of *M. pneumoniae* infection as they are elevated in 50-60% patients. Further, they are also elevated in various other infectious agents, for example Epstein - Barr virus, Cytomegalovirus, *Klebsiella pneumoniae* as well as in the course of malignancies of lymphoid cells and auto-immune diseases.[49]

There are a number of specific serological tests for *M. pneumoniae* that utilize a variety of different methods and antigens. The two most frequently [6] used and widely available are the complement fixation (CF) and enzyme immunoassays (EIAs).

## COMPLEMENT FIXATION METHOD

This method mainly measures the early IgM response and does not differentiate between antibody classes; this is desirable to differentiate acute from remote infection. Complement fixation (CF) suffers from low sensitivity and specificity because glycolipid antigen mixture used may be found in other microorganisms, as well as human tissues and even plants. Cross-reactions[53] with *M. genitalium* are well recognized. Despite these limitations, some Microbiologists still consider a single 1:64 CF titer as an indication of recent *M. pneumoniae* infection.

## ENZYME IMMUNOASSAYS

Enzyme immunoassays (EIAs) are the most widely used and reliable commercial Mycoplasma serology tests. It allows IgG and IgM titration and presents 92% sensitivity and 95% specificity on paired samples. Sero-conversion timing is from three to eight weeks. They are more sensitive than culture for detecting acute infection and can be comparable in sensitivity to polymerase chain reaction (PCR), provided that a sufficient time has elapsed since infection for antibody to develop and patient has a functional immune system. Most EIAs are sold as 96 well microtiter plate formats. However, two EIAs are packaged as qualitative membrane based procedures for the detection of single test specimen. They are rapid (10mn or less) and simple to perform.

1. Immunocard: it measures IgM only
2. Ramel EIA: it detects IgM and IgG simultaneously. The manufacturers have endorsed the use of a single assay for the diagnosis of acute infection in younger persons.

Though the single one point assay appears attractive, conventional plate type EIAs may be more efficient and cost effective in laboratory that needs to measure larger number of specimens at the same time. According to Talkington[4], acute and convalescent sera are necessary for greatest accuracy.

## CULTURE

Culture is laborious, expensive and time-consuming. Compared to PCR, its sensitivity may be no more than 60% even in experienced LAB with strict adherence to procedures.[4] But when positive, its specificity is 100%, provided that appropriate steps are taken to identify the species. The persistence of Mycoplasma for variable periods following acute infection also makes it difficult in some cases to assess the significance of positive culture without additional confirmatory tests such as serology. Therefore, culture is rarely used for routine diagnosis and/or management of *M. pneumoniae* infections. Due to the above mentioned limitations, if it is attempted, it should be augmented by additional diagnostic methods such as PCR and/or serology.[49] Because of the organism's sensitivity to adverse environmental conditions, proper specimen collection, storage, and transport are critical for maintaining viability for culture processing and DNA extraction. Due to its limited metabolic and biosynthetic capacity, it needs extensive nutritional requirements during culture. SP4 medium has become the most successful and widely used broth and agar medium for cultivating *M. pneumoniae* for clinical purposes. Despite the low sensitivity of culture, isolation of organisms has led to some insight into the pathogenesis of extra pulmonary manifestations, because successful isolation provides evidence of direct invasion by viable Mycoplasma.

Sometimes, it is necessary to perform additional tests[19] to conclusively prove that Mycoplasma isolated is indeed *M. pneumoniae*. *M. pneumoniae* differs from other mycoplasma especially from commensals oropharyngeal mycoplasmas, as it has slower growth, ferments glucose, absorbs erythrocytes in the growing colonies, and reduces tetrazolium when grown aerobically or anaerobically.

## ANTIGEN DETECTION TECHNIQUE

Rapid assays for direct antigenic detection of *M. pneumoniae* in respiratory tract specimens:

1. Direct immunofluorescence
2. Counter immunoelectrophoresis
3. Immunoblotting
4. Antigen capture enzyme immunoassay

All these tests suffer from low sensitivity and cross reactivity with other Mycoplasma found in the respiratory tract.

## MOLECULAR BIOLOGY TECHNIQUES

### DNA probes

DNA probes can be used to detect *M. pneumoniae*, the target being 16S rRNA Genes. Disadvantages include the relatively short life span of six weeks, a need for specific equipment, high cost, and the need to purchase and eliminate radioisotopes. They have low sensitivity and specificity [54] and have been replaced by other methods.

### Polymerase chain reaction

Owing to the insensitivity and prolonged time needed for culture and the requirement of collecting acute and convalescent serum at two to three weeks apart for optimal serological diagnosis, Polymerase chain reaction (PCR) has gained considerable interest from the beginning of its inception. There are several advantages of PCR based analysis. First of all, sensitivity of PCR is very high. It has got potential ability to complete the test procedure in a day, so possibility of obtaining a positive result sooner after the onset of illness than serology. Unlike serology, it requires only one specimen. It can provide information about possible mycoplasma etiology in extra pulmonary involvement in which an obvious contribution of respiratory infections may not be apparent readily. Lastly, it does not require viable organisms only. It can amplify the dead bacilli also. PCR may also detect Mycoplasma in tissue processed for histological examination. Since, Nucleic Acid Amplification Techniques (NAATs) targeting DNA can detect both viable and non viable organisms, detecting RNA by reverse transcriptase PCR or nucleic acid sequence based amplification may be a useful method to identify productive *M. pneumoniae* infections.[55]

The various targets that have been used include primarily the ATPase gene, P1 adhesin, 16 S rRNA Gene, tuf gene etc.

Molecular based assays often demonstrate equivalent or superior sensitivity for detection of acute infection over serology as well as culture, this is not always the case.[56] Though, PCR has high sensitivity for the detection of *M. pneumoniae*, serological tests should always be performed to distinguish between acute and persistent infections.[34]

### Positive PCR but negative serology tests

1. Presence of pathogen in the respiratory tract is not necessarily associated with clinical symptoms. Gnarpe[57] *et al*. found that 5.1-13.5% of healthy adults harbor the organisms in the throat. Transient asymptomatic carriage of *M. pneumoniae* results from persistent of the pathogen after disease, and from infections during incubation period. It is currently unknown whether a diagnostic rise in antibody titer regularly occurs in asymptomatic infections.
2. In immuno compromised patients, no diagnostic antibody response may be observed.
3. Early successful antibiotics therapy.

### Positive PCR in a culture-negative person without evidence of respiratory disease

1. Persistence of the organisms after infection.
2. Asymptomatic carriage, perhaps in a intracellular compartment that does not yield culturable organisms.

Negative PCR results in culture or serologically proven infections increase the possibility of inhibitors or other technical problems with the assay and its gene target.[4] Reznikov [58] *et al*. showed that PCR inhibition was more likely to occur with nasopharyngeal aspirates than with throat swabs and recommended the latter specimen for diagnostic purposes. On the other hand, Dorigo-Zetsma [59] *et al*. performed a comprehensive examination with 18 patients with *M. pneumopniae* respiratory tract infection and they found that sputum was the specimen that was most likely to be PCR positive (62.5% versus 41% for nasopharynx, 28% for throat swabs). There are commercial reagents for nucleic acid purification that are effective in removing most inhibitors of amplification in PCR assays.

Combined use of PCR with IgM [6] serology may be a useful approach for diagnosis of *M. pneumoniae* respiratory infection in children, but potentially less useful in adults who may not mount an IgM response. A possible alternative, especially in older adults may be a combination of PCR with IgA serology. Combining these two diagnostic modalities may help in distinguishing colonization from active disease.

In recent time there have been several advancements in PCR technique. Hardegger [60] *et al*. found that a real-time PCR assay was equal to a conventional nested PCR with regard to sensitivity in detection of *M. pneumoniae* in clinical samples, allowing for quantitation of the amplified product during PCR combined with a reduction in hands-on time. Development of quantitative PCR will be beneficial in facilitating better understanding of carrier state associated with *M. pneumoniae*. Loens [61] *et al*. developed a real-time multiplex nucleic acid sequence based amplification (NASBA assay) technique for detection of *M. pneumoniae*, *C. pneumoniae* and Legionella species in respiratory specimens. It is a promising tool for the detection of *M. pneumoniae*, *C. pneumoniae* and Legionella species in respiratory specimens, regarding handling, speed, and number of samples that can be analyzed in a single run, although it is marginally less sensitive than the real-time mono NASBA assay. It definitely calls for further evaluation of large number of clinical samples from CAP patients. Wide spread non-availability of diagnostic techniques for mycoplasma in our country is definitely a great obstacle in elucidating the true prevalence of infection and making timely diagnosis. Though PCR offers improvements in sensitivity, specificity and rapidity over culture and serology, the need remains for the development of cheap, widely available and reproducible diagnostic techniques suitable for our country.

## MANAGEMENT OF *M. PNEUMONIAE* INFECTION

A common view in the treatment of *M. pneumoniae*, especially for mild infections, is that it really does not matter whether anti bacterial is given for most of these infections because the mortality rate is low and these infections are often self limiting and there are confounding effects of mixed infections. Nevertheless, studies [62] from 1960s indicate that treatment for mild *M. pneumoniae* infections reduces the morbidity of pneumonia and shortens the duration of symptoms. In such studies therapy with macrolide or a tetracycline was better than penicillin.

Due to the lack of cell wall, all mycoplasmas are innately resistant to all beta-lactams and glycopeptides. Sulfonamides, trimethoprim, polymyxins, nalidixic acid, and rifampin are also inactive. *M. pneumoniae* is susceptible to antibiotics that interfere with protein or DNA synthesis, such as tetracyclines, macrolides, and quinolones. Macrolides are the most active agents in vitro and azithromycin is the most active macrolide with minimum inhibitory concentrations ranging from 0.0003 to 0.031mg/ml.[63] Azithromycin is preferred over erythromycin due to better side-effects profile and once-a-day dose. Therapy with macrolide antibiotics can also reduce the rate of recurrent wheezing and abnormal pulmonary function that result from acute *M. pneumoniae* infection. Azithromycin may also be effective in the prevention of infection with *M. pneumoniae* infection during outbreak.

In a Japanese study,[64] Telithromycin, a ketolide antibiotic was found having good activity against 41 clinical isolates of *M. pneumoniae*. The authors determined the *in vitro* activity of telithromycin and found it to be less potent than azithromycin but it was more active than four other macrolides (erythromycin, clarithromycin, roxithromycin, josamycin), levofloxacin and minocycline. Its MICs at which 50% and 90% of the isolates were inhibited were both 0.00097mg/ml, justifying further clinical studies to determine its efficacy for treatment of *M. pneumoniae*. The quinolones [65] are cidal *in vitro* and several quinolones, including levofloxacin, gatifloxacin, moxifloxacin, gemifloxacin are highly active against *M. pneumoniae* *in vitro*. In generals, quinolones appear to be slightly less active than macrolides *in vitro*. However, activity *in vitro* does not always predict microbiological activity *in vivo*. Fluroquinolones have been shown to be bactericidal for *M. pneumoniae*, whereas macrolides and tetracyclines are primarily bacteriostatic.

A study based on the microtitre susceptibility testing method by Duffy[65] *et al*. demonstrates excellent potency of Gemifloxacin *in vitro* with MICs range for mycoplasma ≤ 0.008 to 0.125mg/l. Gemifloxacin was found as potent as or more potent than tetracycline, clindamycin and other quinolones investigated.

In children,[66] only macrolides can be safely used regardless of age because of the potential side effects of tetracyclines and quinolones in younger patients. Principi[67] *et al*. in a study including 191 children hospitalized for CAP with evidence of acute *M. pneumonmiae* or *C. pneumoniae* found that 106(97.2%) of the 109 children treated with macrolides and only 67(81.7%) of the 82 treated with other antibiotics were considered cured or improved after four to six weeks (*P* value<0.05).

Recently, focus of interest has been on antimicrobial resistance in *M. pneumoniae*. Macrolide resistance was reported to emerge in Japan.[68,69] It can be easily selected *in vitro*. Such mutants typically [4] exhibit the macrolide-lincosamides-streptogramin B type resistance, rendering lincosamides and streptogramin B inactive in addition to macrolides. It is well established that *M. pneumoniae* developed macrolide resistance by point mutations leading to A-to-G transitions in the peptidyl transferase loop of domain V of the 23S rRNA gene at positions 2063 and 2064, which reduces the affinity of macrolide for the ribosome. The likelihood of *M. pneumoniae* developing resistance to macrolides by this mechanism under natural conditions may be enhanced, since there is only a single rRNA[70] operon in the *M. pneumoniae* genome. Currently, identification of these resistant strains relies on time consuming and labor intensive procedures such as restriction fragment length polymorphism, MIC studies, and sequence analysis.

Wolff [71] *et al*. describe a real-time based PCR and high resolution melt analysis for rapid detection of macrolide resistant strain in *M. pneumoniae*. Fluoroquinolones[72] may show anti mycoplasmal activities against macrolide and tetracycline resistant strains of *M. pneumoniae* because of their mechanism of action and their excellent penetration into lung tissue, in particular bronchial secretions.

However, despite macrolide resistance, clinical failure is unlikely. Suzuki[73] *et al*. compared clinical outcomes in 11macrolide resistant and 26 macrolide susceptible patients were given macrolide therapy. The resistant group showed more febrile days during initial macrolide therapy than susceptible patients. But, no apparent treatment failure or serious illness was reported for macrolide resistant patients. It can be explained by the immuno modulatory effect of macrolide.

An investigational agent Cethromycin also showed excellent activity against *M. pneumoniae* *in vitro* with MICs lower [74] than those of macrolides. It belongs to the ketolide family, a new class of antibiotic derived from macrolide. However, there are no *in vivo* studies involving this agent against *M. pneumoniae*. So far, no consensus on the duration of therapy with macrolides has been reached, and treatment schemes spanning from one to three weeks have been described. The most widely used are: azithromycin 10mg/kg/day, a daily dose not exceeding 500mg /dose for five days and clarithromycin 15mg/kg/day divided into two doses, not exceeding 500mg/dose for 10-15days_4_.

Some recent studies[75,76]have suggested that the addition of antibiotic prophylaxis to standard epidemic control measures in contacts may be useful during institutional outbreaks of *M. pneumoniae* pneumonia. However, when deciding whether to use mass prophylaxis in closed settings, many factors including the induction of antibiotic resistance, cost, allergic reactions etc must be considered.

Corticosteroids may be beneficial in *M. pneumoniae* CNS disease,[77] but experience with this therapy is limited to case reports and small series. Plasma exchange has been reported to be effective in transverse myelitis and polyradiculitis. Administration of intravenous immune globulins can also be considered.

### Vaccination

*M. pneumoniae* is a leading cause of both upper and lower respiratory tract infections that can lead to devastating sequela like neurological complications. In addition, there is lack of natural protective immunity following primary infection and also infection with *M. pneumoniae* is associated with prolonged carriage, and a propensity for outbreaks in military camps, schools, and hospitals. [4] Development of a vaccine also seemed to be promising in view of the facts that the organism is rather homogeneous antigenically and there appears to be some protection against re-infection. So an effective vaccine against *M. pneumoniae* is desirable because it would not only prevent severe disastrous outcome of infection such as encephalitis but also reduce milder illnesses causing distress and loss of workdays particularly among soldiers, school children and health care workers. In a recent meta analysis[78] of six clinical trials with a total of 67,268 subjects, the efficacy of the vaccines against *M. pneumoniae* specific pneumonia ranged between 42% and 54% when diagnosis was performed by culture and serology respectively. The summarized efficacy of *M. pneumoniae* vaccine against pneumonia, regardless of etiologies, was 36%. No significant adverse reactions (including autoimmune effects) were observed in this meta-analysis. The inactivated *M. pneumoniae* vaccines may reduce the total rates of both pneumonia and respiratory infections by ~40%. Therefore, there is definitely a need for redeveloping *M. pneumoniae* vaccines both for high-risk settings as well as for general population.

## CONCLUSION

Advances in detection and characterization of *M. pneumoniae* by using PCR, serology, and culture augmented by knowledge obtained from the complete genome sequence of this organism, has led to the appreciation of its role as a human pathogen. Despite these many advances, much is still unknown about this tiny bacterium, which is among the smallest of all free living forms. Most mycoplasma infections in clinical settings never have a microbiological diagnosis because rapid, sensitive, specific, and reasonably priced methods are not readily available in many clinical settings.

Practically, serological tests are the only means by which *M. pneumoniae* infections are diagnosed on a wide scale, and this method has a number of limitations. A reliable and user-friendly amplified or non-amplified method for detection of the mycoplasma or its nucleic acid in clinical specimens would be of immense importance for patient diagnosis and management, for furthering knowledge of a potential role in chronic lung diseases.
